# BIGapp: A user‐friendly genomic tool kit identified quantitative trait loci for creeping rootedness in alfalfa (*Medicago sativa* L.)

**DOI:** 10.1002/tpg2.70067

**Published:** 2025-07-10

**Authors:** Alexander M. Sandercock, Michael D. Peel, Cristiane H. Taniguti, Josué Chinchilla‐Vargas, Shufen Chen, Manoj Sapkota, Meng Lin, Dongyan Zhao, Arlyn J. Ackerman, Bhoja R. Basnet, Craig T. Beil, Moira J. Sheehan

**Affiliations:** ^1^ Breeding Insight Cornell University Ithaca New York USA; ^2^ USDA‐ARS Forage and Range Research Lab Utah State University Logan Utah USA

## Abstract

Alfalfa (*Medicago sativa* L.) is a globally vital forage crop valued for its perennial growth and multiple annual harvests. A breeding effort is underway to improve the crop for productivity and persistence against biotic and abiotic stresses using “creeping rootedness,” a trait where plants exhibit horizontal root growth, similar to rhizomes, with increased vegetative ground surface area. In this study, we genotyped a breeding population of 648 alfalfa lines segregating for creeping rootedness using the 3K DArTag marker panel to identify trait‐associated genomic loci and evaluate the feasibility of genomic prediction to accelerate breeding cycles. Using genome‐wide association studies (GWAS), we identified three quantitative trait loci (QTLs), with one major QTL located on chromosome 6.1 associated with this trait. Genomic prediction showed moderate predictive ability (*r* = 0.68) for creeping rootedness. A significant advancement in this study was the development and utilization of the Breeding Insight Genomics Application (BIGapp), an R Shiny application designed to streamline the processing of genomic data through an intuitive interface. This tool makes integrating genomics into existing breeding programs accessible, regardless of species ploidy or the researcher's coding proficiency. The identified QTL will be essential in future efforts to develop new alfalfa cultivars with the creeping rootedness trait and accelerate the breeding cycle, with BIGapp playing a pivotal role in these advancements.

## Core Ideas

- Breeding Insight Genomics Application (BIGapp) is a user‐friendly tool kit that simplifies genomic analyses.
- Evaluated the genetic architecture for creeping rootedness in alfalfa with BIGapp.
- Identified three quantitative trait loci associated with the alfalfa creeping rootedness trait.
- Genomic prediction shows a moderate predictive ability for creeping rootedness.

**Abbreviations**
BIGapp: Breeding Insight Genomics Application
BIGr: Breeding Insight Genomics R Package
BLUE: best linear unbiased estimate
DArT: diversity arrays technology
GS: genomic selection
GT: genotype
GWAS: genome‐wide association study
LD: linkage disequilibrium
MADC: missing allele discovery counts
MAF: minor allele frequency
MAS: marker‐assisted selection
PC: principal component dimension
PCA: principal component analysis
QTL: quantitative trait locus
RRID: research resource ID
SNP: single‐nucleotide polymorphism
USDA: united states department of agriculture
VCF: variant call file

## 1 INTRODUCTION

Alfalfa (*Medicago sativa* L.) is one of the most extensively grown forage crops, with between 32 and 40 million ha under cultivation globally (Castonguay et al., 2006; Luo et al., 2019; Russelle, 2001). It has tremendous agronomic importance due to its high nutritional value for cattle, perennial growth, and nitrogen‐fixing capabilities (Flajoulot et al., 2005; Mårtensson & Ljunggren, 1984). In primarily grass pastures, forage production is most often limited by available soil N (Chinea & Arévalo, 2014; Solomon et al., 2011). Supplemental N fertilizer is an added expense or not feasible for most rangeland situations. Low bioavailable soil N can be mitigated by including N‐fixing legumes into rangelands and grazing situations, which has been shown to increase productivity and more uniform distribution of seasonal forage production (Cox et al., 2017; Sleugh et al., 2000). More specifically, the value of alfalfa in grazing lands has been well‐documented for many years, as alfalfa inclusion boosts forage yield and subsequent increases in animal mass (Berdahl et al., 1989; Cox et al., 2017; Sleugh et al., 2000; Waldron et al., 2020). Despite the benefits alfalfa provides for forage animal nutrition, even the hardiest improved cultivars still succumb to decreases in plant stands under grazing, which is exacerbated and hastened on arid rangelands (Heinrichs, 1954; Misar et al., 2015; Van Keuren & Matches, 1988).

Typical hay‐type *M. sativa* alfalfa cultivars have a single deep taproot with narrow or protruding crowns, resulting in limited tolerance to animal trampling under grazing, as well as frost injury, which increases their susceptibility to biotic and abiotic stresses and consequently leads to poor stand persistence (Brummer & Bouton, 1991; Kehr et al., 1963). Traits important to survival and persistence are variously expressed in different alfalfa cultivars and may include broad or deep crowns, spreading or creeping rootedness, and dormant types in drought‐prone areas. The use of creeping rootedness is supported by Heinrichs, who demonstrated that new shoots from horizontal roots, for example, the creeping root trait, protect the plants from mortality due to trampling during grazing as well as protection from winter injury (Heinrichs, 1975). This was further demonstrated by the release of Travois, a very hardy dormant alfalfa with a proliferating root system (Rumbaugh et al., 1965).

Commercially cultivated purple‐flowered alfalfa (*M. sativa* subsp. *sativa*) is a self‐incompatible cross‐pollinated autotetraploid (2*n* = 4*x* = 32) (McCoy & Bingham, 1988). There are both diploid (2*n* = 2*x* = 16) and tetraploid yellow‐flowered forms of *M. sativa*, which are classified as subsp. *Falcata*, while the purple‐flowered forms are classified as subsp. *sativa* (Brummer et al., 1999). The *falcata*‐type alfalfa genetic background is not found in high proportions in commercial cultivars. However, it remains a valuable source of germplasm for improved persistence due to winter, drought, and grazing tolerance, as well as the spreading/creeping root phenotype (Berdahl et al., 1986; Rumbaugh,^1965^).

The genetic basis of the creeping root phenotype is largely unknown and understudied (Heinrichs, 1954; Polegri et al., 2011). A lack of genetic studies for creeping rootedness has hindered its incorporation by marker‐assisted selection (MAS), genomic selection (GS), or other screening approaches aimed at expediting the breeding process (e.g., seedling screening and speed breeding). Genomic resources for tetraploid alfalfa include several chromosomal‐scale reference genomes (H. Chen et al., 2020; Li et al., 2020; Shen et al., 2020), a 9K single‐nucleotide polymorphism (SNP) marker array (X. Li et al., 2014), and a diversity arrays technology (DArT) 3K DArTag panel, which is a targeted amplicon genotyping platform (X. Zhao, Han, et al., 2023; D. Zhao, Mejia‐Guerra, et al., 2023). The 3K DArTag panel has already shown its power to identify quantitative trait loci (QTLs) and evaluate breeding populations (Medina, Heuschele, et al., 2024; Medina, Zhao, et al., 2024). This panel provides a rapid, affordable, and reproducible method to evaluate genome‐wide markers across different genotyping projects, and it has been shown to be effective in both diploid and tetraploid alfalfa species (D. Zhao et al., 2024).

Despite the increasing number of genomic resources for alfalfa, many breeders face barriers to implementing genomic tools into their programs. The barriers include the cost and time to genotype (GT), uncertainty or incomplete adoption of routine genotyping into their breeding cycle effectively, and coding expertise needed to analyze the large and complex data formats of next‐generation sequencing technologies. When breeding programs lack in‐house expertise, they must rely on external collaborators, hire bioinformaticians, or purchase services from genotyping vendors. This can lead to added costs, delays in analyzing results for time‐sensitive breeding decisions, or vendors using improper bioinformatic tools designed for model diploid species. R Shiny applications exist to leverage the strengths of the R coding language while providing a more user‐friendly way (i.e., low‐ or no‐code) to view and analyze data in a web‐based environment (Chang et al., 2024). Recent R Shiny applications exist for analyzing RNAseq data (Farrel et al., 2023), visualizing genomic data (T. Chen et al., 2023; Ramirez et al., 2020), and analyzing genome‐wide identity‐by‐descent data (Mahmoudiandehkordi et al., 2024). However, many of these applications primarily support diploid species, posing an additional challenge for researchers working with autopolyploids, which are more commonly encountered by specialty crop breeders. Thus, an R Shiny application that can provide the ability to preprocess and analyze genomic data, especially read depth data from targeted genotyping platforms, regardless of the ploidy of the target organism, would lower the barrier for breeders to efficiently and reproducibly integrate genomics into their existing breeding programs to assist in making breeding decisions.

Here, we introduce the Breeding Insight Genomics R Shiny application (BIGapp, research resource ID [RRID]: SCR_026676) and breeding insight genomics R package (BIGr) (RRID: SCR_026677, available on CRAN (https://CRAN.R‐project.org/package=BIGr). The BIGapp is designed to offer accessible, web‐based genomic and bioinformatics tools tailored for analyzing both diploid and autopolyploid data. Using BIGapp, we analyzed 648 alfalfa samples that were sequenced with the alfalfa 3K DArTag panel to explore the genomic basis of creeping rootedness in a mapping population. Specifically, we (i) established a breeding population of alfalfa exhibiting the creeping‐rooted phenotype, characterized by lateral root spreading; (ii) identified QTLs associated with creeping rootedness; and (iii) evaluated the potential of GS for this trait. These findings aim to enhance alfalfa breeding programs through the identification of QTL related to creeping rootedness, and the BIGapp enables researchers to analyze and publish their genomic data with various ploidy levels regardless of their computer coding background.

**Core Ideas**

- Breeding Insight Genomics Application (BIGapp) is a user‐friendly tool kit that simplifies genomic analyses.
- Evaluated the genetic architecture for creeping rootedness in alfalfa with BIGapp.
- Identified three quantitative trait loci associated with the alfalfa creeping rootedness trait.
- Genomic prediction shows a moderate predictive ability for creeping rootedness.

## 2 MATERIALS AND METHODS

### 2.1 Breeding population design

**FIGURE 1:**
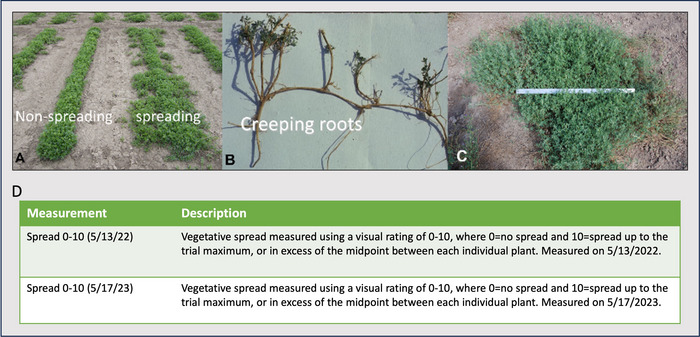
The creeping rooted phenotype in alfalfa grown at the study site near Logan, UT. (A) Seeded rows of non‐spreading versus spreading alfalfa showing early spring growth. (B) Creeping root structure showing multiple shoots arising from the single creeping root. (C) A 9‐year‐old single alfalfa plant showing spread in comparison to a meter stick. (D) The descriptions for each of the two measured traits used to evaluate creeping rootedness.

A spreading/creeping rooted, tetraploid alfalfa population developed at the United States Department of Agriculture (USDA) Agricultural Research Service Forage and Range Research unit in Logan, UT, was the parental source of spreading phenotype. This material was selected specifically for its spread and survival/persistence under dry‐range conditions typical of the Intermountain Region of the Western United States. It was selected from a broad genetic base of cultivars (released prior to 1982), experimental lines, and naturalized populations that included both *sativa* and *falcata* types exhibiting the creeping rooted phenotype. The *falcata* background was derived primarily from germplasm introduced by N.E. Hansen as described by Boe et al. (2020). Two plants from this population were used as the spreading parents and designated Entry 3 and Entry 8. These plants were chosen as parents for their strong expression of the creeping root/spreading phenotype (Figure 1). The two spreading plants were vegetatively cloned to generate additional plants for crossing. The plants with the spreading phenotype were manually crossed in a greenhouse with six plants from a non‐spreading commercial‐type *sativa* (ID = CT3‐08). Seed was collected from both the spreading (Entry 3 and Entry 8) and non‐spreading (CT3‐08) parents in each cross and used for testing.

The different crosses within the spreading root mapping population were assigned a color name. Each color corresponds to a specific cross between spreading and non‐spreading alfalfa parental lines (Figure S1). Green designates progeny from reciprocal crosses between the two spreading parental lines (Entries 3 and 8), resulting in full‐sibling progenies. Blue, pink, and red are three separate cross events between the spreading parent (Entry 3) and the non‐spreading parent (CT3‐08), whereas orange, white, and yellow represent crosses between the spreading parent (Entry 8) and the non‐spreading parent (CT3‐08) (Figure S1). All progeny within each of these crosses are full siblings.

Field plots were located at the Utah State University Evans Research Farm near Logan, UT (41°41′36″ N, 111°49′55″ W, elevation 1381 m). Soil at the Evans Farm is a Nibley silty clay loam (fine, mixed, mesic Aquic Argiustolls) where annual precipitation averages 47 cm (1981–2010) (http://www.usclimatedata.com, November 8, 2024). The Evans Farm site is in the Central Great Basin of the western United States, characterized by hot, dry summers where most of the annual precipitation typically occurs as snowfall.

Plant materials were established as transplanted spaced plants in 10‐plant plots in the spring of 2014. Plants were started in the greenhouse the second week of January in Ray Leach Cone‐tainers (Stewe and Sons) and transplanted to the field in the second week of May. Dead plants were replaced with plants from their respective cross 3 weeks after transplanting. Plants were established on 1‐m centers within rows with 1‐m spacing between rows. The 1‐m spacing between all plants allowed for easy measurement of spread from any individual plant. Prior to planting, mono‐ammonium phosphate (11N‐52P‐0K) was applied at 90 kg ha^−1^ providing 9.9 and 46.6 kg ha^−1^ each of N and P, respectively. The application was made to ensure plant P requirements were met for the duration of the study.

The field experiment was conducted using an augmented incomplete block design, with repeated checks and parental lines distributed across the experimental field, which was divided into four incomplete blocks. Individual plants were arranged at the intersections of rows and columns, each being treated as an independent observational unit.

### 2.2 Data collection

Vegetative spread was assessed at two different time points, May 13, 2022 and May 17, 2023, during the study using a visual rating scale of 0–10, where 0 = no spread and 10 = spread up to the trial maximum, or in excess of, the midpoint between each individual plant (Figure 1). Spread from single plants was rarely uniform, as a single plant might spread excessively in one orientation but not in another. To accommodate this, the spread was measured between the two widest points on the same plant. In some instances, the determination of spread was subjective. As the plants matured, their crowns grew wider than is common in stands with low plant density, a condition intentionally created for this study. Therefore, it was possible to classify a plant as spreading/creeping rooted when it was simply a large crown. However, these cases would always appear at the low end of the spreading spectrum.

### 2.3 Generation of best linear unbiased estimates (BLUEs)

Phenotypic data were analyzed with a two‐phase mixed effects model in ASReml‐R (Butler et al., 2018). To ensure unbiased GT estimates suitable for genome‐wide association studies (GWAS), GT was modeled as a fixed effect to avoid over shrinkage (Holland & Piepho, 2024), and the two‐phase analysis was utilized to accommodate unbalanced, multi‐environment data (Endelman, 2023). Experiments were conducted across two consecutive years (2022 and 2023), with each year being treated as a unique environment throughout the analysis.

In the first phase, GT effects were designated explicitly as fixed effects to derive BLUEs separately for each year and accurately quantify GT‐specific performance unbiased by shrinkage, through the following fixed effects model:

$$y_{ijk}=\mu +G_{i}+S_{jk}+\varepsilon_{ijk}$$

where *y_ijk_* is the observed phenotype for the *i*th GT in the *j*th column and *k*th row, *μ* is the overall mean, *G_i_* denotes the fixed effects of GTs, and *S_jk_* is a random spatial effect associated with the plot at column *j* and row *k*, which is modeled as below:

*S_jk_* follows a normal distribution *N* (0, Σ_spatial_) where *Var*(*S_jk_*) = Σ_spatial_ = [(*σ* ^2^_c_ Σ_c_) ⊗ Σ_r_]. Here, *σ* ^2^_c_ is the column variance, Σ_c_ is the first‐order autoregressive‐correlation matrix with elements {*σ*_mn_} = *ρ* ^|m‐n|^ for plots in columns m and n (in the same row), and Σ_r_ is the first‐order autoregressive matrix for rows. The symbol ⊗ implies the Kronecker product of the two auto‐correlation matrices. *ρ* is the autocorrelation parameter for both row and column factors. *ε_ijk_* is the residual error, which is independently and identically distributed with mean 0 and variance *σ* ^2^. BLUEs were estimated for each GT within each environment, and precision weights were applied for each estimate as follows:

$$w_{il}=\frac{1}{SE_{il}^{2}}$$

where $w_{il}$ is the weight for the *i*th GT in the *l*th environment and $SE_{il}^{2}$ is the corresponding standard error. BLUEs were carried into phase 2 as the response variable in the following precision‐weighted fixed effect model:

$$y_{il}=\mu +G_{i}+\varepsilon_{il}$$

where *y_il_* is the BLUE for the *i*th GT in the *l*th environment, *μ* is the overall mean, *G_i_* is the fixed effect of the *i*th GT, and $\varepsilon_{il}$ is the weighted residual error term, modeled with variance as follows:

$$\mathrm{Var}\;\left(\varepsilon_{il}\right)=\frac{\sigma^{2}}{w_{il}}$$

where $\mathrm{Var}(\varepsilon_{il})$ is the variance of the weighted residual for the *i*th GT in the *l*th environment, and $\sigma^{2}$ is fixed at one, implying that the precision weight (*w_il_*) determines residual variance $\mathrm{Var}(\varepsilon_{il})$ (Supporting Information 1).

Broad‐sense heritability (*H* ^2^) for each of the two creeping root measurements was calculated independently using the getHeritability() function from the spatial analysis of field trials with splines R package (v1.0.19; Oakey et al., 2006; Rodríguez‐Álvarez et al., 2018) as implemented in the MrBean R Shiny application (v2.0.9; Aparicio et al., 2023).

### 2.4 Sequencing and processing of genomic data

A previously developed alfalfa 3000 DArTag marker panel (D. Zhao, Mejia‐Guerra, et al., 2023) was used to GT the 648 alfalfa individuals. DArT provided a missing allele discovery counts (MADC) file that contains the 81 bp amplicon at each of the 3000 panel target loci and their multiallelic information (Supporting Information 2). From this, we extracted the read count information for the target 3000 SNPs and for the additional genetic variants (off‐target SNPs) contained within the 81 bp region using the BIGapp “Convert to VCF” tab (VCF is variant call file) (Table 1). The “Convert to VCF” tab relies on the BIGr madc2vcf_all() function, which performs pairwise alignments between all of the unique sequence reads (microhaplotype) and the reference to obtain all the biallelic SNPs contained within each microhaplotype using the R/Rsamtools package (v2.22.0; Morgan et al., 2025). In the process, sequences with low alignment score (<40) and multiallelic SNPs are discarded. Alternatively, DArT can provide a VCF file of target and off‐target SNPs that is derived from DArT's internal SNP calling pipeline, upon request. The read count information for a total of 6172 target and off‐target SNPs was converted to a VCF file format and then used as the initial input for BIGapp “Dosage Calling” tab (Table 1).

**TABLE 1:** The Breeding Insight Genomics Application (BIGapp) modules and the supporting breeding insight genomics R package (BIGr) functions were used to preprocess and analyze the alfalfa creeping root population.

Tool	Module/function	Description
Genotype processing		
BIGapp	Convert to VCF	Convert genotype data to the standard variant call format (VCF) file. This module includes the ability to extract biallelic SNPs from the DArTag MADC format.
BIGapp	Dosage calling	Use read count information in a VCF or MADC file format to generate genotype calls using the R/Updog package, output the information in VCF format.
BIGapp	VCF filtering	VCF files can be filtered to remove low quality SNPs and samples.
BIGr	madc2vcf_all()	Function to extract the read count information for the target and off‐target biallelic SNPs contained within the DArTag MADC file. The output is a VCF file.
BIGr	updog2vcf()	Function to convert the output from the R/Updog dosage calling *multidog()* function to a VCF format (Gerard, 2023; Gerard & Ferrão, 2020).
BIGr	filterVCF()	Function to filter a VCF file for SNP and sample quality metrics.
Summary metrics		
BIGapp	Genomic diversity	Calculate SNP minor allele frequency, sample observed heterozygosity, dosage call ratios, and visualize marker distribution.
BIGr	calculate_Het()	Calculate the observed heterozygosity for each sample.
Population structure		
BIGapp	PCA	Evaluate genomic relationships between samples using a principal component analysis.
GWAS		
BIGapp	GWASpoly	Perform genome wide association analyses to identify quantitative trait loci (QTLs) using the R/GWASpoly package (Rosyara et al., 2016).
Genomic Prediction		
BIGapp	Predictive ability	Estimate the predictive ability of a genomic prediction model using a fivefold cross validation. The estimated breeding values (EBVs) and predicted traits can be estimated for samples with and without phenotype information.

*Note*: All modules/functions listed here support diploid and polyploid data. Both the BIGapp and BIGr contain additional functions for processing genotypic and phenotypic breeding data not described here.

Abbreviations: GWAS, genome‐wide association study; PCA, principal component analysis; SNP, single‐nucleotide polymorphism.

Dosage calls were estimated from the read count information in the BIGapp “Dosage Calling” tab. The read count information was first extracted from the VCF file with the vcfR package (v1.15.0) and converted to matrices (*m* × *n*), where *m* is equal to the number of samples and *n* is equal to the number of SNPs (Knaus & Grünwald, 2017). These read count matrices were used by the updog R package (v2.1.5) with the “norm” population model and ploidy level of four to estimate dosage calls for each of the alfalfa samples (Gerard, 2023; Gerard & Ferrão, 2020; Gerard et al., 2018). The resulting VCF file contained the estimated updog dosage calls, dosage calling statistics, and read count information.

The VCF file of raw dosage calls was then filtered in the BIGapp “VCF Filtering” tab. The R/updog genotyping quality metrics were first used to remove SNPs if they had an allelic bias (Bias) value below 0.2 or >2, a locus‐specific estimated overdispersion parameter (OD) value >0.05, posterior proportion of individuals GTd incorrectly for that SNP (Prop_mis) >0.05, and a maximum posterior probability that the GT estimate for the SNP/sample is correct (maxpostprob) <0.9 (BIGr v0.5.5; https://CRAN.R‐project.org/package=BIGr). SNPs were removed with a minor allele frequency (MAF) threshold <0.05. The dosage value was set to missing if the read depth for any marker‐sample combination was <10. Finally, SNPs and samples were removed if they had ≥50% missing data. The filtered GT dosage file contained 2211 SNPs for 648 samples (Supporting Information 3).

### 2.5 Summary metrics and structure of GT data

Summary metrics for the filtered alfalfa genotyping data were evaluated with the BIGapp “Genomic Diversity” tab. MAF was estimated for each SNP. Observed heterozygosity and the GT distributions were estimated per sample. The genomic relationship between each sample was visualized with a principal component analysis (PCA) in the “PCA” tab. The filtered VCF file was input, and the information from the GT field was extracted with vcfR (v1.15.0) and then converted to a matrix of numeric GT values of the alternate allele count (0 = homozygous reference, and 4 = homozygous alternate) (Knaus & Grünwald, 2017). This GT matrix was then converted to an additive genomic relationship matrix with the Gmatrix function from the AGHmatrix R package (v2.1.4) with the VanRaden parameter (Amadeu et al., 2023). This relationship matrix was then used by prcomp() from base R to calculate the eigenvectors. The PCA was then visualized with R packages plotly (v4.10.4; Li & Bilal, 2021; Wickham, 2011) for a three‐dimensional visualization and ggplot2 (v3.5.1; Li & Bilal, 2021; Wickham, 2011) for a two‐dimensional figure.

### 2.6 QTL analysis

A genome‐wide association study (GWAS) was performed to first identify SNPs associated with the creeping root phenotype using the BIGapp “GWASpoly” tab. We implemented the GWASpoly R package (Rosyara et al., 2016) for its ability to use both polyploid and diploid species data. The input VCF file was first converted to the GWASpoly input format, where SNP location information is in the first two columns of a GT matrix. BLUEs for the creeping rootedness trait were used for the phenotype information. As outcrossing species may potentially be pollinated by contaminated pollen, 417 progenies (Supporting Information 1) with known pedigree and expected GT cluster patterns (Figure S2) were selected for GWAS. The mixed linear model for GWAS included principal components (PCs) based on SNP GT data and a kinship matrix to control for variation in population stratification and unequal relatedness, respectively. PC and the kinship matrix were calculated using GWASpoly (v2.13; Rosyara et al., 2016). The optimal model for GWAS was selected based on the Bayesian information criterion (Schwarz, 1978) for the trait (Figure S3A). For tetraploid alfalfa, different genetic models were tested, including additive, dominant (“1‐dom” and “2‐dom”), general, diplo‐additive, and diplo‐general. The threshold for significance was the “M.eff” method for its balance of computational efficiency and accuracy. The *p*‐value distribution from GWAS was visualized using a *Q*–*Q*‐plot (Figure S3B) with code derived from the CMplot package (LiLin‐Yin, 2024).

A genome‐wide linkage disequilibrium (LD) decay plot was used to determine the minimum distance (8.575 Mb) between genetically independent markers (*r* ^2^ < 0.05; Figure S4). Based on the selected window size, the significant markers identified were further evaluated for their location within 8.575 Mb LD regions with the get.QTL function and percent of explainable variance for the trait of interest using the fit.QTL function from GWASpoly as implemented in BIGapp (Rosyara et al., 2016). The final set of QTL from the GWASpoly additive model for each trait was then evaluated for their proximity to genes from the XinJiangDaYe *M*. *sativa* reference genome annotation (H. Chen et al., 2020; D. Zhao, Mejia‐Guerra, et al., 2023).

### 2.7 Genomic prediction

Predictive ability of a genomic prediction model was estimated for the creeping rootedness trait in the BIGapp “Predictive Ability” tab. For the same set of 417 samples used in GWAS, GT information was extracted from the VCF files and converted into a GT matrix. The GT matrix was then normalized and scaled so that the dosage calls were within a −1 to 1 range for compliance with the A.mat() function. The A.mat() function from ridge regression best linear unbiased prediction (rrBLUP) (v4.6.3) was used to generate an additive relationship matrix, where missing data were imputed with the mean value of each SNP (Endelman, 2011). A fivefold cross‐validation with the kin.blup() function was then performed for 10 iterations (Endelman, 2011). The predictive abilities were the averaged Pearson correlation coefficients between the predicted breeding value and BLUEs of the creeping root phenotype across 10 iterations.

## 3 RESULTS AND DISCUSSION

### 3.1 Phenotype and GT summary for the creeping root breeding population

**FIGURE 2:**
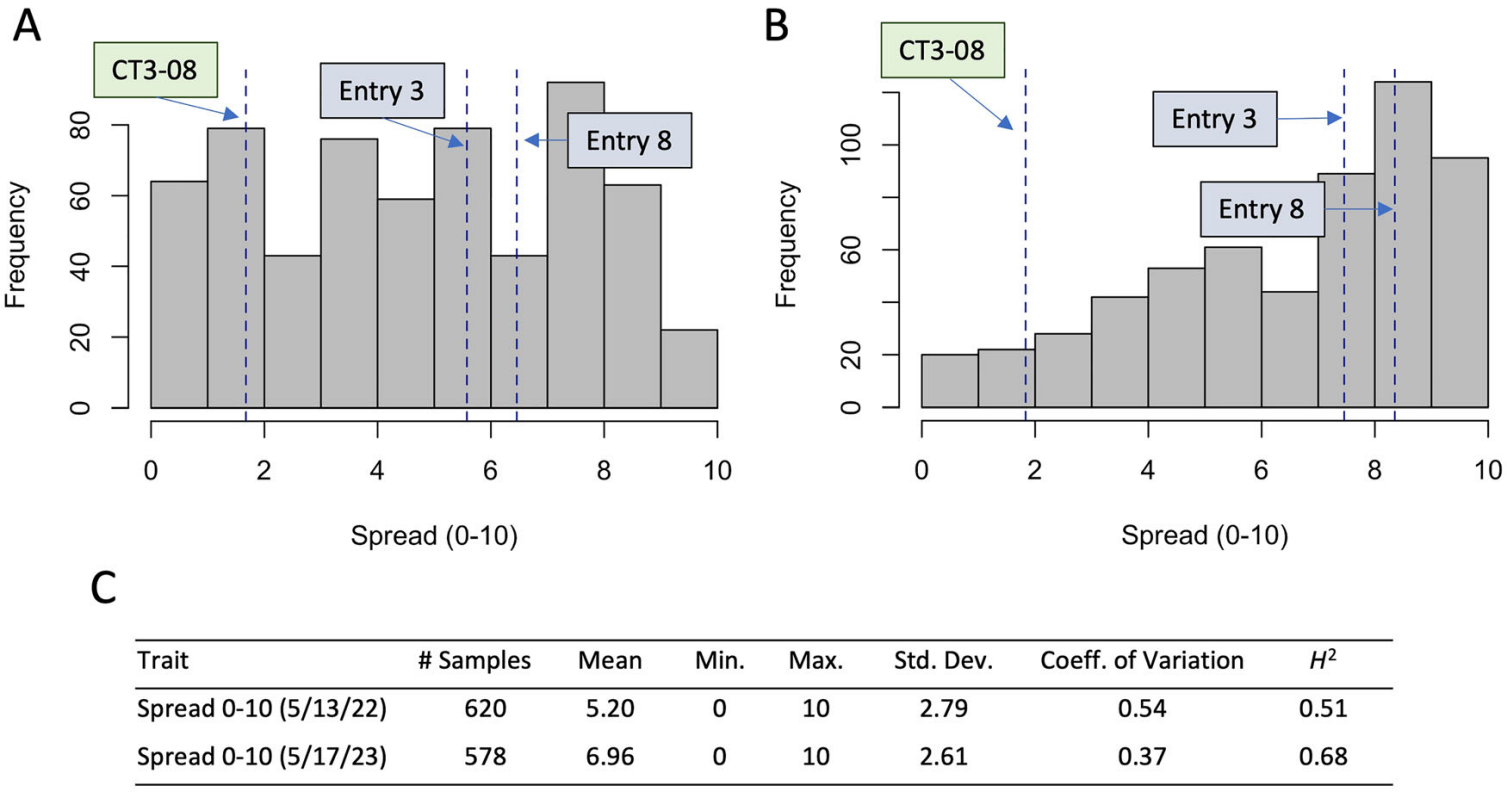
Summary of two measurements for creeping rootedness across all phenotyped samples. (A) Distribution of the ratings for “Spread 0–10 (5/13/22).” (B) Distribution of the ratings for “Spread 0–10 (5/17/23).” (A–B) The mean value for the spreading parental lines (Entry 3 and Entry 8) and the non‐spreading parental line (CT3‐08) is labeled with vertical dashed lines. (C) Summary metrics for the two creeping root measurements.

The mean values for the non‐spreading alfalfa parental line (CT3‐08) were consistently below the average across all samples for both measurements (Figure 2). Both spreading parental lines had above‐average measurements for each trait, with Entry 8 recording greater mean values than Entry 3 (Figure 2). BLUEs were generated from the raw trait datasets to account for spatial variance within the planting areas. There was a high correlation between the two spread traits, with a Pearson's correlation value of 0.8. Broad‐sense heritability was higher for the Spread 0–10 measurement taken on 5/17/23 (*H* ^2^ = 0.68) compared to the measurement from May 13, 2022 (*H* ^2^ = 0.51) (Figure 2C).

A total of 2211 SNPs across 648 samples remained after filtering for GT data quality. Genome‐wide SNP detection was largely well distributed, apart from Chromosome 6.1, which showed lower SNP density (1.7 SNPs/Mb vs. genome‐wide mean = 3.24 SNPs/Mb) (Figure S5B; Table S1), which was also present in the raw sequencing data (Figure S5A). Population summary metrics found that most SNPs contained at least one alternative allele, while homozygous reference alleles were the second most common GT. The average minor‐allele frequency across all markers was 0.249 (Figure S6A,B). Consistent with the prevalence of heterozygous SNPs, the average observed heterozygosity for the alfalfa samples was 0.63 (Figure S6C).

### 3.2 Structure of the creeping rooted breeding population

**FIGURE 3:**
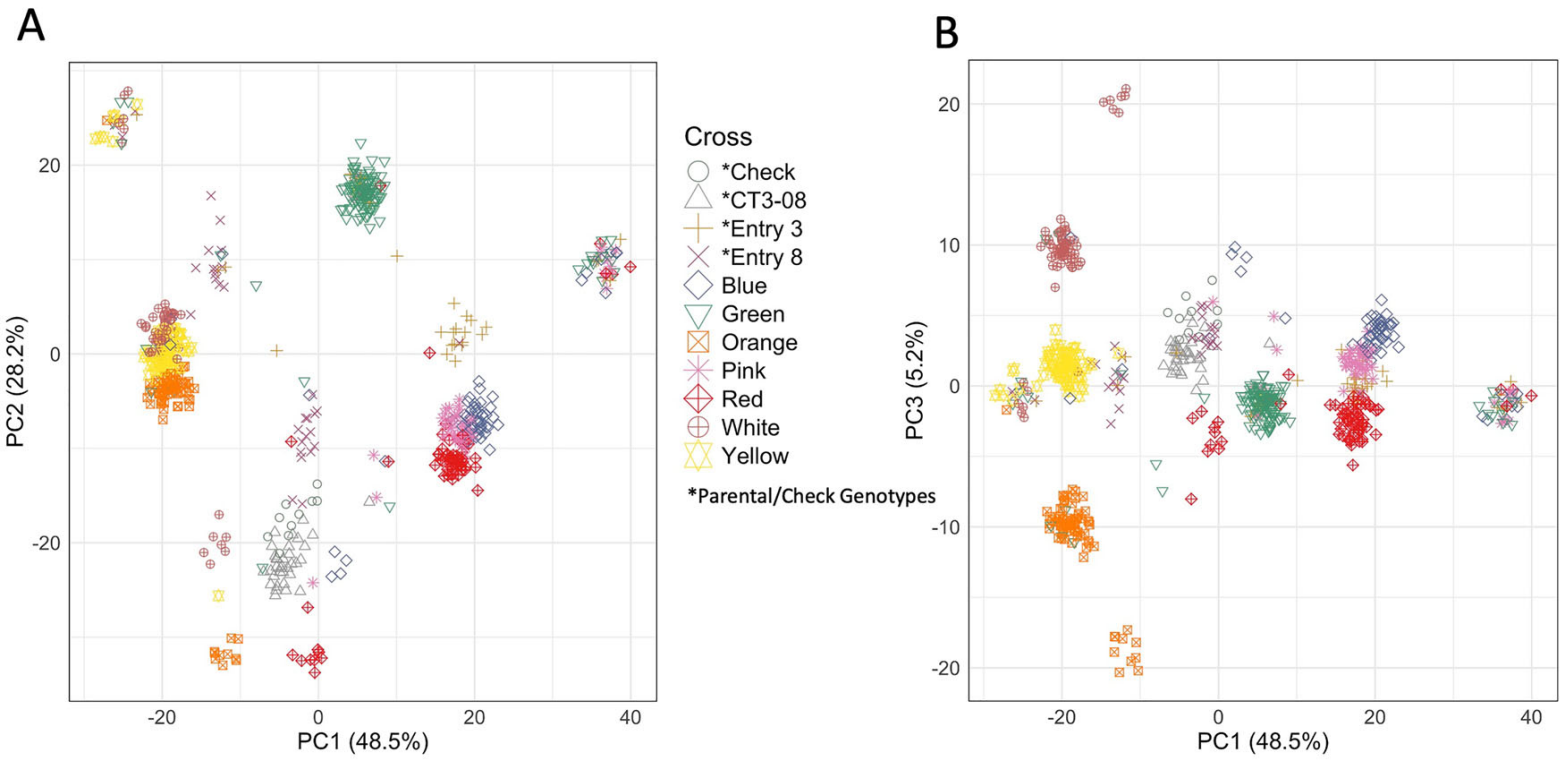
Principal component analysis of the alfalfa creeping root breeding population (*n* = 648). (A) Genetic relations plotted for PC1 and PC2 dimensions. (B) Genetic relations plotted for PC1 and PC3 dimensions. Each point on the plot is an individual plant, and the colors/shapes are indicative of their parental cross. The labels denoted with “*” were the parental/check plants. The progeny derives from the following crosses: Orange, White, and Yellow (Entry 8 × CT3‐08); Blue, Pink, and Red (Entry 3 × CT3‐08); and Green (Entry 3 × Entry 8). (A) PC1 versus PC2 and (B) PC1 versus PC3.

Population structure within the breeding population was visualized with a PCA to evaluate the relationships between individuals depending on their parental crosses. Moderate clustering due to the parental cross shows three distinct clusters, with the Green progeny being one, the Orange, Yellow, and White progeny being another, and the final distinct cluster containing the Red, Pink, and Blue cross progeny (Figure 3). This clustering pattern matches the spreading parental GT (either Entry 3 or Entry 8), with a mixture of both Entry 3 and Entry 8 progeny in the Green cross cluster, and the parental GTs distributed between the clusters (Figure 3A). The Yellow, Orange, and White progeny separate into distinct clusters when comparing PC1 to PC3 (Figure 3B). The remaining samples did not belong to an identifiable cluster based on parental cross (Figure 3), which could be potentially caused by pollen contamination when making crosses.

### 3.3 QTL and candidate genes associated with creeping root

**FIGURE 4:**
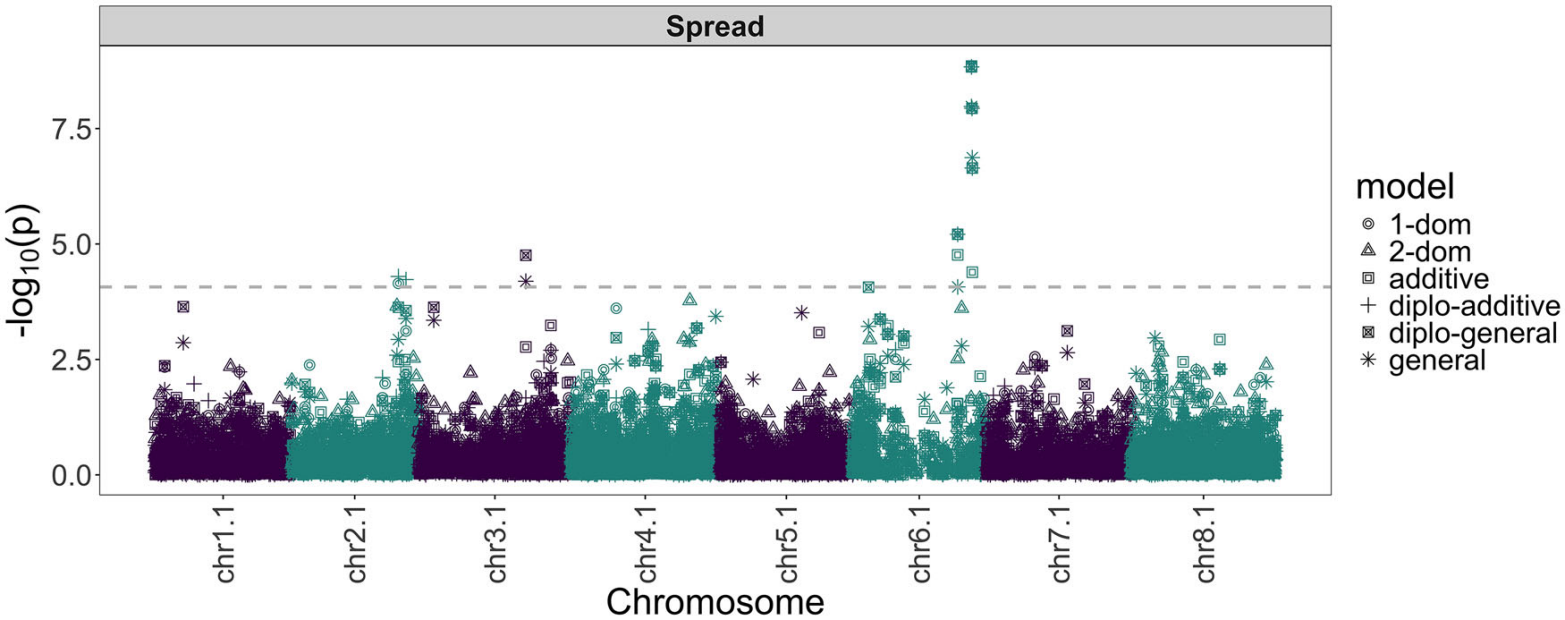
Manhattan plot for the creeping root trait across all genome‐wide association study (GWAS) models. 2210 single‐nucleotide polymorphisms (SNPs) were evaluated with 417 progeny using GWASpoly. The M.eff significance threshold is the horizontal dashed line. SNPs above that line were significantly associated with the creeping root trait.

A GWAS analysis was performed to identify genomic regions significantly associated with the creeping rooted trait. The filtered 2211 SNPs and 417 selected samples were used in the association analysis (Figure S2). Using the phenotypic BLUE values, eight unique SNPs (Table S2) were found to be associated with the creeping root trait using six genetic effect models. A basic local alignment search tool analysis of microhaplotypes (54–81 bp in length) harboring significant spread trait‐associated SNPs revealed that the sequence harboring chr6.1_016478433 had a single copy in each of the four sub‐genomes, but these copies were not located in syntenic regions. Additionally, chr6.1_016478433 showed moderate LD (*r* ^2^ = 0.41) with the peak SNP (chr6.1_071635255) near the terminus of Chromosome 6.1, indicating potential misplacement of chr6.1_016478433. Therefore, chr6.1_016478433 was removed from the GT data, and the GWAS analysis was refined accordingly (Figure 4).

Application of an 8.575 Mb LD‐pruning window identified three distinct QTLs across five GWAS models (Table 2). These include one major QTL located on Chromosome 6.1 and two minor QTLs: one on Chromosome 2.1 (1‐dom model) and another on Chromosome 3.1 (diplo‐general model) (Table 2). The major QTL on Chromosome 6.1 was significant in all GWAS models except the 2‐dom model (Table 2) and explained 9.09% of the phenotypic variance in the breeding population under the additive model. Progeny (*n* = 263) that were homozygous of the reference allele (GGGG) at the peak SNP (chr6.1_071635255) showed an average spreading root rating of 5.53, while progeny (*n* = 153) with one alternative allele (GGGA) showed an average rating of 8.12, indicating that the alternative allele is favorable for the creeping root trait. The peak SNP was located within a gene annotated as exonuclease 1 (MS.gene58914). Examination of the coding sequence revealed that the SNP occurs in the third exon, resulting in a synonymous mutation (CTG to TTG) that maintains leucine as the encoded amino acid.

**TABLE 2:** Quantitative trait loci (QTL) associated with alfalfa creeping rootedness.

Marker	Model	Threshold	Chrom	Position	Ref	Alt	Score	Effect
chr6.1_071635255	Additive	4.47	chr6.1	71,635,255	0	1	8.85	1.260
chr6.1_071635255	General	4.47	chr6.1	71,635,255	0	1	7.98	NA
chr3.1_065262400	Diplo‐general	4.47	chr3.1	65,262,400	0	1	4.76	NA
chr6.1_071635255	diplo‐general	4.47	chr6.1	71,635,255	0	1	8.84	NA
chr6.1_071635255	diplo‐additive	4.47	chr6.1	71,635,255	0	1	8.84	1.277
chr6.1_071635255	1‐dom‐alt	4.4	chr6.1	71,635,255	0	1	8.84	1.277
chr2.1_064883289	1‐dom‐ref	4.07	chr2.1	64,883,289	0	1	4.15	−1.077

*Note*: Significant markers from each of the evaluated traits from GWASpoly were filtered to remove linked markers within an 8.575 Mb window, and the most significant marker in each linkage block was retained. Markers were filtered for linkage disequilibrium (LD) separately within each GWASpoly genetic model.

Abbreviations: Alt, alternative allele; Ref, reference allele.

### 3.4 GS with the creeping root traits

The predictive ability of a fivefold cross‐validation selection model was estimated to determine the feasibility of using GS for creeping rootedness. We found that creeping rootedness had a moderate predictive ability with a mean value of 0.68 (Figure S7).

## 4 DISCUSSION

The goals of this study were to evaluate the creeping root trait in alfalfa to identify QTL and evaluate the performance of this trait for GS. To achieve this, we genotyped 648 alfalfa samples from a creeping root breeding population using the alfalfa 3K DArTag panel, resulting in 2211 high‐quality SNPs (including both target and off‐target SNPs). From the 648 genotyped alfalfa plants, 417 progeny were selected based on their clustering in a PCA for GWAS and GS analyses. We identified three QTLs that were significantly associated with creeping rootedness, with one major QTL that explained 9% of the phenotypic variance. GS showed a moderate predictive ability with a mean value of 0.68. To facilitate these analyses, we developed the Breeding Insight Genomics R Shiny Application (BIGapp) to provide a coding‐free way for breeders to evaluate genomic data and assist in making selection decisions.

### 4.1 Creeping root breeding population

We found moderate levels of observed heterozygosity among the alfalfa samples and a medium average (0.249) MAF among the filtered markers. Compared to wild accessions, originating from Central Asia, northeastern Europe, Balkans‐Turkey‐Black Sea, and Siberia/Mongolia, within the USDA National Plant Germplasm System, the average heterozygosity of the creeping root breeding population was more than twice the average observed heterozygosity of each of these Eurasian alfalfas (Medina, Zhao, et al., 2024). This is likely due to our sampled population intentionally maintaining higher diversity through use of both the *M. sativa* subsp. *sativa* and *M. sativa* subsp. *falcata* types. Marker distribution was genome‐wide with a deficiency of SNPs on Chromosome 6.1 in both the raw and filtered datasets. This is likely due to a known limitation of the panel design, as there is sparse marker coverage on the short arm near the centromere of Chromosome 6.1, and similar gaps are present in a previous alfalfa study that used the DArTag panel (D. Zhao et al., 2024).

The PCA analysis showed moderate clustering based on the parental crosses, with the parental and check GTs distributed between the progeny clusters. Most of the progeny with shared parentage clustered within the same groups, suggesting that population structure is influenced by the genetic background of the parental lines. However, several outliers among the progeny deviated from the major progeny clusters based on putative pedigree and were subsequently excluded from the GWAS and GS analysis.

### 4.2 QTL and their associated creeping root genes

Three QTLs were found to be significantly associated with creeping root in a GWAS using an intercross population originating from three parental lines. One major QTL was identified on Chromosome 6.1 with the alternative allele (A) of the peak SNP (chr6.1_071635255) as the favorable allele, showing elevated allele frequency in both spreading parents (Entry 3 and Entry 8). Initial GWAS analysis identified chr6.1_016478433 as a minor‐effect QTL in addition to the major QTL on Chromosome 6. However, LD analysis revealed a moderate correlation (*r* ^2^ = 0.41) between chr6.1_016478433 and chr6.1_071635255, suggesting these two markers likely belong to the same LD block. The position of chr6.1_016478433 may be incorrectly placed due to genome assembly challenges in the first alfalfa sub‐genome. Given this positional uncertainty and our current resources, we decided to exclude chr6.1_016478433 in the second round of GWAS.

The peak SNP (chr6.1_071635255) of the major QTL is located within the putative creeping root gene, MS.gene58914, which was found to be expressed in root tissue in an alfalfa RNAseq study (Miller et al., 2022), supporting that this gene could influence the differential growth in creeping root alfalfa. Exonucleases are essential enzymes that play critical roles in cellular metabolism and genome stability by cleaving DNA from free ends, supporting various cellular maintenance processes. Although no direct link between exonuclease 1 (MS.gene58914) and root growth has been established in the current literature, a reduction in this gene could increase mutation rates during DNA transcription. The SNP in exonuclease 1 does not alter the amino acid sequence, but the codon change from CTG to TTG could significantly impact translation efficiency. Studies in rice have shown a strong codon usage bias, with the CTG codon for leucine being six times more prevalent than TTG in actively translating ribosome complexes, implying that the TTG codon may have lower translation efficiency (Zhao et al., 2017). While further investigation into this gene and its mutants would be necessary to determine its potential role in creeping rootedness in alfalfa populations, its association with the trait may still be valuable for breeding purposes and allele tracking in crosses.

In addition to identifying the gene within which the peak QTL was located, we also report the associated genes that were within the 8.575 Mb linkage window of the three QTLs (Supporting Information 4). However, exploration of all the associated genes within the QTL window is beyond the scope of this paper. As such, any errors in unannotated regions from the reference genome would lead to undiscovered SNP‐gene associations. It should be noted that while we identify candidate QTL and genes associated with creeping root in alfalfa, we may not have located the causal QTL or genes due to the reduced genome representation of the mid‐density DArTag panel. Rather, we have identified candidate regions for further exploration or used them for targeted breeding decisions to introgress the creeping rootedness trait. Future studies into the identified regions with a significant association with the creeping root trait should focus on (1) fine mapping of the major Chromosome 6.1 QTL to narrow down the region and identify the causal genes and (2) validation of the putatively causal gene through gene expression, overexpression, or knockout experiments. These follow‐up analyses can validate the regions that influence the creeping root trait, leading to a more targeted breeding effort and a better understanding of this phenotype.

### 4.3 Implications for creeping root in alfalfa breeding and genomic prediction

The QTL we found related to creeping root could be used to inform future alfalfa breeding efforts and expedite breeding cycles. Our finding of a major QTL (and two additional minor QTLs) that contribute to the creeping root trait in this intercross population suggests that this trait can be incorporated into other breeding populations quickly. This integration of these QTLs into existing breeding programs allows for the simultaneous selection of the creeping root trait alongside other desirable traits, such as flowering date and yield (Adhikari et al., 2019; He et al., 2020; Zhang et al., 2019), ultimately leading to the development of improved elite cultivars. Furthermore, the identification of these candidate QTLs enables MAS as a practical approach to improve selection decisions and screening tools in alfalfa (Li & Brummer, 2012).

In this work, we found that using a genomic best linear unbiased prediction GS model revealed a moderate predictive ability for the creeping root trait. We employed a fivefold cross‐validation method for estimating the predictive ability of the creeping root trait, and this could likely be optimized using alternative models, testing different marker filtering options, and weighting trait‐associated markers (Alemu et al., 2024; Heslot et al., 2012; Medina et al., 2021). Our use of a mid‐density DArTag panel could have led to slightly conservative estimates for predictive ability, but is likely comparable to using higher density genotyping methods (Simeão Resende et al., 2014; VanRaden, 2020). We have demonstrated that GS is suitable for predicting the phenotype of alfalfa samples for the creeping root trait, particularly since it is likely polygenic. Future iterations of BIGapp will include support for additional GS methods. Our results demonstrate that the GS implementation in BIGapp currently can streamline the breeding process, ultimately contributing to the development of improved alfalfa cultivars with desirable traits.

### 4.4 Utility of BIGapp for data analysis

BIGapp was developed to lower the barriers of integrating genomics into breeding programs and provide accessible genomic pipelines for breeding decisions. As shown in this study, its key feature is the support of both diploid and polyploid species, allowing breeders to preprocess, analyze, and visualize genomic data without requiring coding skills. Importantly, each analysis tab is independent, allowing users to utilize VCF files generated from various pipelines or platforms as long as they contain GT or read count information. Alternatively, breeders can process genomic information within BIGapp and export it as a VCF file for use in other pipelines. Many of the custom functions within BIGapp are also available in the BIGr R package, catering to experienced R users who want to incorporate these functions into their own bioinformatic pipelines. Comprehensive training materials, including step‐by‐step tutorials and in‐app guides, are provided to enhance user engagement and facilitate the interpretation of results. The development of BIGapp represents a significant advancement in making genomic data analysis accessible to non‐experts in coding across diverse species and ploidy levels. By packaging bioinformatic pipelines in a reproducible and user‐friendly interface, BIGapp promotes standardized approaches for analyzing diploid and polyploid breeding data in a centralized location.

## 5 CONCLUSION

In summary, the alfalfa creeping root trait is crucial for alfalfa populations, as it enhances persistence over the long term when facing grazing pressures and thereby increases productivity. We have demonstrated that one major QTL predominantly influences the creeping root trait in this alfalfa intercross population and that its moderate predictive ability makes it feasible to use GS to augment alfalfa breeding progress. The markers used in this study were obtained from the alfalfa 3K DArTag panel and can be reproducibly sequenced in other alfalfa populations. Lastly, the BIGapp R Shiny application and BIGr R package lower the barriers for integrating genomics into breeding programs regardless of the species’ ploidy or breeder's coding expertise.

## AUTHOR CONTRIBUTIONS

**Alexander M. Sandercock**: Conceptualization; data curation; formal analysis; methodology; software; visualization; writing—original draft; writing—review and editing. **Michael D. Peel**: Conceptualization; data curation; formal analysis; investigation; methodology; resources; supervision; writing—original draft; writing—review and editing. **Cristiane H. Taniguti**: Data curation; formal analysis; methodology; software; validation; visualization; writing—review and editing. **Josué Chinchilla‐Vargas**: Formal analysis; investigation; software; validation; visualization. **Shufen Chen**: Data curation; formal analysis; methodology; software; validation; visualization. **Manoj Sapkota**: Data curation; formal analysis; methodology; resources; software; supervision; validation; visualization. **Meng Lin**: Formal analysis; investigation; project administration; software; supervision; validation; visualization. **Dongyan Zhao**: Investigation; methodology; project administration; software; supervision; validation; visualization; writing—review and editing. **Arlyn J. Ackerman**: Data curation; formal analysis; methodology; validation. **Bhoja R. Basnet**: Data curation; formal analysis; methodology; validation; visualization. **Craig T. Beil**: Project administration; resources; supervision; writing—review and editing. **Moira J. Sheehan**: Funding acquisition; project administration; resources; supervision; writing—review and editing.

## CONFLICT OF INTEREST STATEMENT

The authors declare no conflicts of interest.

## Supporting information




**Supplemental Table 1**: Summary of the distribution of the filtered 2,211 SNPs.
**Supplemental Table 2**: Significant markers associated with alfalfa creeping rootedness.
**Supplemental Figure 1**: Summary of the genetic backgrounds of 800 plants evaluated for creeping rootedness.
**Supplemental Figure 2**: PCA with the progeny labelled for their inclusion in the GWAS and GS analysis.
**Supplemental Figure 3**: GWAS diagnostic plots.
**Supplemental Figure 4**: Linkage‐disequilibrium decay plot for the filtered markers.
**Supplemental Figure 5**: Distribution of the alfalfa SNPs.
**Supplemental Figure 6**: Genomic diversity of the alfalfa creeping root breeding population.
**Supplemental Figure 7**: Box plot of the 10 iterations for the GS GBLUP model evaluation.

**Supplemental File 1**: Raw phenotypic and BLUE data for the alfalfa samples.

**Supplemental File 2**: Raw DArTag MADC File.

**Supplemental File 3**: Filtered VCF file of the 2,211 biallelic SNPs and 648 samples.

**Supplemental File 4**: Genes associated with the three GWAS identified QTL based on a 8.575 Mb LD window.

## Data Availability

Genomic and phenotypic data files are included as Supporting Information: BLUE calculations: https://github.com/Breeding‐Insight/alfalfa_BLUE; BIGr (RRID: SCR_026677): https://CRAN.R‐project.org/package=BIGr; BIGapp (RRID: SCR_026676): https://github.com/Breeding‐Insight/BIGapp. BIGapp is available on Sci‐Net for USDA‐ARS scientists.
