# Supplementary material for: BIGapp: A user‐friendly genomic tool kit identified quantitative trait loci for creeping rootedness in alfalfa (Medicago sativa L.)
Source: Plant Genome. 2025 Jul 10;18(3):e70067. doi: 10.1002/tpg2.70067 (PMC12245737; doi:10.1002/tpg2.70067)
Supplement: Supplementary file 1 [file TPG2-18-e70067-s003.docx]

**Supplemental material**

**BIGapp: A User-Friendly Genomic Tool Kit Identified Quantitative Trait Loci for Creeping Rootedness in Alfalfa (*Medicago sativa* L.)**

Alexander M. Sandercock^*^, Michael D. Peel^*^, Cristiane H. Taniguti, Josué Chinchilla-Vargas, Shufen Chen, Manoj Sapkota, Meng Lin, Dongyan Zhao, Arlyn J. Ackerman, Bhoja R. Basnet^1^, Craig T. Beil, Moira J. Sheehan

The supplemental material includes two tables, seven figures, and four files, which provide the following information:

Supplemental Table 1: Summary of the distribution of the filtered 2,211 SNPs.

Supplemental Table 2: Significant markers associated with alfalfa creeping rootedness.
Supplemental Figure 1: Summary of the genetic backgrounds of 800 plants evaluated for creeping rootedness.

Supplemental Figure 2: PCA with the progeny labelled for their inclusion in the GWAS and GS analysis.

Supplemental Figure 3: GWAS diagnostic plots.

Supplemental Figure 4: Linkage-disequilibrium decay plot for the filtered markers.

Supplemental Figure 5: Distribution of the alfalfa SNPs.

Supplemental Figure 6: Genomic diversity of the alfalfa creeping root breeding population.

Supplemental Figure 7: Box plot of the 10 iterations for the GS GBLUP model evaluation.

Supplemental File 1: Raw phenotypic and BLUE data for the alfalfa samples.

Supplemental File 2: Raw DArTag MADC File.

Supplemental File 3: Filtered VCF file of the 2,211 biallelic SNPs and 648 samples.

Supplemental File 4: Genes associated with the three GWAS identified QTL based on a 8.575 Mb LD window.

**Supplemental Table 1. Summary of the distribution of the filtered 2,211 SNPs mapped to the XinJiangDaYe alfalfa reference genome.**

| **Chromosome** | **Length (bp)** | **# SNPs** | **SNP Density (SMP/Mb)** |
| --- | --- | --- | --- |
| Chr1.1 | 82459472 | 271 | 3.286463 |
| Chr2.1 | 76462061 | 250 | 3.269595 |
| Chr3.1 | 93149498 | 286 | 3.070333 |
| Chr4.1 | 90245664 | 376 | 4.166405 |
| Chr5.1 | 81211777 | 296 | 3.644792 |
| Chr6.1 | 80303593 | 137 | 1.706026 |
| Chr7.1 | 88407277 | 282 | 3.189783 |
| Chr8.1 | 87242343 | 313 | 3.587707 |

**Table S2. Significant markers associated with alfalfa creeping rootedness.** Results from each of the evaluated traits from the six GWASpoly genetic models that had significance scores higher than the M.eff threshold.

| **Marker** | **Model** | **Threshold** | **Chrom** | **Position** | **Ref** | **Alt** | **Score** | **Effect** |
| --- | --- | --- | --- | --- | --- | --- | --- | --- |
| chr6.1_016478433 | additive | 4.47 | chr6.1 | 16478433 | 0 | 1 | 6.61 | 1.09268872 |
| chr6.1_063320970 | additive | 4.47 | chr6.1 | 63320970 | 0 | 1 | 4.71 | 0.67052423 |
| chr6.1_071635255 | additive | 4.47 | chr6.1 | 71635255 | 0 | 1 | 8.74 | 1.25737664 |
| chr6.1_071635271 | additive | 4.47 | chr6.1 | 71635271 | 0 | 1 | 8.74 | 1.25737664 |
| chr6.1_071935194 | additive | 4.47 | chr6.1 | 71935194 | 0 | 1 | 7.83 | 1.20574283 |
| chr6.1_016478433 | general | 4.47 | chr6.1 | 16478433 | 0 | 1 | 6.61 | NA |
| chr6.1_071635255 | general | 4.47 | chr6.1 | 71635255 | 0 | 1 | 7.87 | NA |
| chr6.1_071635271 | general | 4.47 | chr6.1 | 71635271 | 0 | 1 | 7.87 | NA |
| chr6.1_071935194 | general | 4.47 | chr6.1 | 71935194 | 0 | 1 | 7.83 | NA |
| chr6.1_072112174 | general | 4.47 | chr6.1 | 72112174 | 0 | 1 | 6.75 | NA |
| chr3.1_065262400 | diplo-general | 4.47 | chr3.1 | 65262400 | 0 | 1 | 4.75 | NA |
| chr6.1_016478433 | diplo-general | 4.47 | chr6.1 | 16478433 | 0 | 1 | 6.61 | NA |
| chr6.1_063320970 | diplo-general | 4.47 | chr6.1 | 63320970 | 0 | 1 | 5.13 | NA |
| chr6.1_071635255 | diplo-general | 4.47 | chr6.1 | 71635255 | 0 | 1 | 8.72 | NA |
| chr6.1_071635271 | diplo-general | 4.47 | chr6.1 | 71635271 | 0 | 1 | 8.72 | NA |
| chr6.1_071935194 | diplo-general | 4.47 | chr6.1 | 71935194 | 0 | 1 | 7.83 | NA |
| chr6.1_072112174 | diplo-general | 4.47 | chr6.1 | 72112174 | 0 | 1 | 6.53 | NA |
| chr6.1_016478433 | diplo-additive | 4.47 | chr6.1 | 16478433 | 0 | 1 | 6.61 | 1.09268872 |
| chr6.1_063320970 | diplo-additive | 4.47 | chr6.1 | 63320970 | 0 | 1 | 5.13 | 0.9095465 |
| chr6.1_071635255 | diplo-additive | 4.47 | chr6.1 | 71635255 | 0 | 1 | 8.72 | 1.27451921 |
| chr6.1_071635271 | diplo-additive | 4.47 | chr6.1 | 71635271 | 0 | 1 | 8.72 | 1.27451921 |
| chr6.1_071935194 | diplo-additive | 4.47 | chr6.1 | 71935194 | 0 | 1 | 7.83 | 1.20574283 |
| chr6.1_072112174 | diplo-additive | 4.47 | chr6.1 | 72112174 | 0 | 1 | 6.53 | 1.09612957 |
| chr6.1_016478433 | 1-dom-alt | 4.4 | chr6.1 | 16478433 | 0 | 1 | 6.61 | 1.09268872 |
| chr6.1_063320970 | 1-dom-alt | 4.4 | chr6.1 | 63320970 | 0 | 1 | 5.13 | 0.9095465 |
| chr6.1_071635255 | 1-dom-alt | 4.4 | chr6.1 | 71635255 | 0 | 1 | 8.72 | 1.27451921 |
| chr6.1_071635271 | 1-dom-alt | 4.4 | chr6.1 | 71635271 | 0 | 1 | 8.72 | 1.27451921 |
| chr6.1_071935194 | 1-dom-alt | 4.4 | chr6.1 | 71935194 | 0 | 1 | 7.83 | 1.20574283 |
| chr6.1_072112174 | 1-dom-alt | 4.4 | chr6.1 | 72112174 | 0 | 1 | 6.53 | 1.09612957 |
| chr2.1_064883289 | 1-dom-ref | 4.07 | chr2.1 | 64883289 | 0 | 1 | 4.13 | -1.0754236 |

Supplemental Figure 2. Simplified summary of the genetic backgrounds of 800 plants evaluated for creeping rootedness

Supplemental Figure 2. Principal component analysis of the filtered 2,211 SNPs and 648 alfalfa genotypes. The individual dots on the plot represent alfalfa samples, and the colors delineate the 417 alfalfa progeny selected for the GWAS and GS analysis (Y = included, N = excluded).

Supplemental Figure 3. GWAS diagnostic plots. A) Bayesian Information Criterion plot to estimate the number of PCs to use in the GWASpoly analysis. In this case, three PCs were used. B) QQ-plots to evaluate the expected and observed p-values of each of the GWASpoly models.

Supplemental Figure 4. Linkage-disequilibrium decay plot for the filtered markers. At 8.575 Mb window size (red line), there is low association between markers (R^2^ < 0.05).

Supplemental Figure 5. Distribution of the alfalfa SNPs. A) The distribution of the raw VCF 6,172 target and off-target SNPs and B) the distribution of the filtered VCF 2,211 SNPs.

Supplemental Figure 6. Genomic diversity of the alfalfa creeping root breeding population. The filtered 2,211 SNPs and 648 samples were evaluated for A) minor-allele frequency distribution, B) percentage of each dosage call across samples, and C) observed heterozygosity per sample. Here, the dosage calls represent the count of alternate alleles, where 0 = homozygous reference, and 4 = homozygous alternate (B).

Supplemental Figure 7. Box plot of the 10 iterations of five-fold cross-validation GBLUP for the creeping root trait. The mean value across the 10 iterations was 0.676.
